# Distribution of estimated glomerular filtration rate and determinants of its age dependent loss in a German population-based study

**DOI:** 10.1038/s41598-021-89442-7

**Published:** 2021-05-13

**Authors:** Thomas Waas, Andreas Schulz, Johannes Lotz, Heidi Rossmann, Norbert Pfeiffer, Manfred E. Beutel, Irene Schmidtmann, Thomas Münzel, Philipp S. Wild, Karl J. Lackner

**Affiliations:** 1 Institute of Clinical Chemistry and Laboratory Medicine, University Medical Center of the Johannes Gutenberg University Mainz, Langenbeckstrasse 1, 55131 Mainz, Germany; 2 Preventive Cardiology and Preventive Medicine, Center for Cardiology, University Medical Center of the Johannes Gutenberg University Mainz, Mainz, Germany; 3 Department of Ophthalmology, University Medical Center of the Johannes Gutenberg University Mainz, Mainz, Germany; 4 Department of Psychosomatic Medicine and Psychotherapy, University Medical Center of the Johannes Gutenberg University Mainz, Mainz, Germany; 5 Institute of Medical Biostatistics, Epidemiology and Informatics, University Medical Center of the Johannes Gutenberg University Mainz, Mainz, Germany; 6 Center for Cardiology - Cardiology I, University Medical Center of the Johannes Gutenberg University Mainz, Mainz, Germany; 7 Center for Thrombosis and Hemostasis, University Medical Center of the Johannes Gutenberg University Mainz, Mainz, Germany; 8 DZHK (German Centre for Cardiovascular Research), Partner Site RhineMain, Mainz, Germany

**Keywords:** Nephrology, Risk factors

## Abstract

Glomerular filtration rate (GFR) declines with age by approx. 1 ml/min/m^2^ per year beginning in the third decade of life. At 70 years of age > 40 ml/min/m^2^ of GFR will be lost. Thus, factors affecting loss of GFR have significant public health implications. Furthermore, the definition of chronic kidney disease based on GFR may not be appropriate for the elderly. We analyzed factors affecting absolute and relative change of eGFR over a 5 year period in 12,381 participants of the Gutenberg Health Study. We estimated GFR at baseline and after 5 years of follow-up by two different equations. Association with the decline of estimated GFR (eGFR) was assessed by multivariable regression analysis. We confirmed a median loss of eGFR per year of approx. 1 ml/min/m^2^. Aside from albuminuria systolic blood pressure was most strongly associated with faster decline of eGFR followed by echocardiographic evidence of left ventricular diastolic dysfunction and reduced ejection fraction. White blood cell count showed a moderate association with eGFR loss. Diastolic blood pressure, serum uric acid and serum albumin were associated with slower GFR decline in multivariable analysis. Sensitivity analysis with exclusion of individuals taking diuretics, antihypertensive, antidiabetic, or lipid lowering drugs confirmed these associations.

## Introduction

Glomerular filtration rate (GFR) is long known to decline beginning in young adulthood. The average loss is approx. 1 ml/min/1.73 m^2^ per year which is mainly due to the loss of functioning nephrons^1,2^. GFR < 60 ml/min/1.73 m^2^ is usually considered as a criterion for chronic kidney disease (CKD) even though additional criteria apply and other definitions have been proposed for epidemiologic studies^3,4^. In any case, it is obvious that with the current life expectancy many people will fall below the threshold of 60 ml/min/1.73 m^2^ during their lifetime. Accordingly, this definition has been challenged recently and it is perhaps not appropriate in the elderly^5,6^. On the other hand the continuous loss of GFR carries the risk that kidney function will become significantly impaired in elderly individuals, if the loss is greater than average. Therefore, risk factors for an increased annual decline of kidney function have major implications for public health in ageing societies.

Several observational studies have focused on risk factors for the development of CKD or terminal renal disease^7–12^. The definitions of CKD were not always the same, but usually included an estimated GFR (eGFR) < 60 ml/min/1.73 m^2^, a decline of GFR of more than 50% within the observation period (usually more than 5 years), or a rapid decline of GFR of more than 3 ml/min/1.73 m^2^ per year. Other studies have analyzed factors which affect the yearly rate of loss of GFR in persons without known kidney disease^13–18^.

Several risk factors for a more than average decline of GFR have been proposed including obesity, chronic inflammatory states, certain coagulation markers, hypertension and others. However, not all proposed risk factors have been consistently validated in independent studies. We analyzed a broad spectrum of biochemical and clinical traits and their association to the decline of GFR over 5 years in the Gutenberg Health Study, a German population based cohort of persons aged 35–74 years at the time of inclusion.

## Results

**Figure 1:**
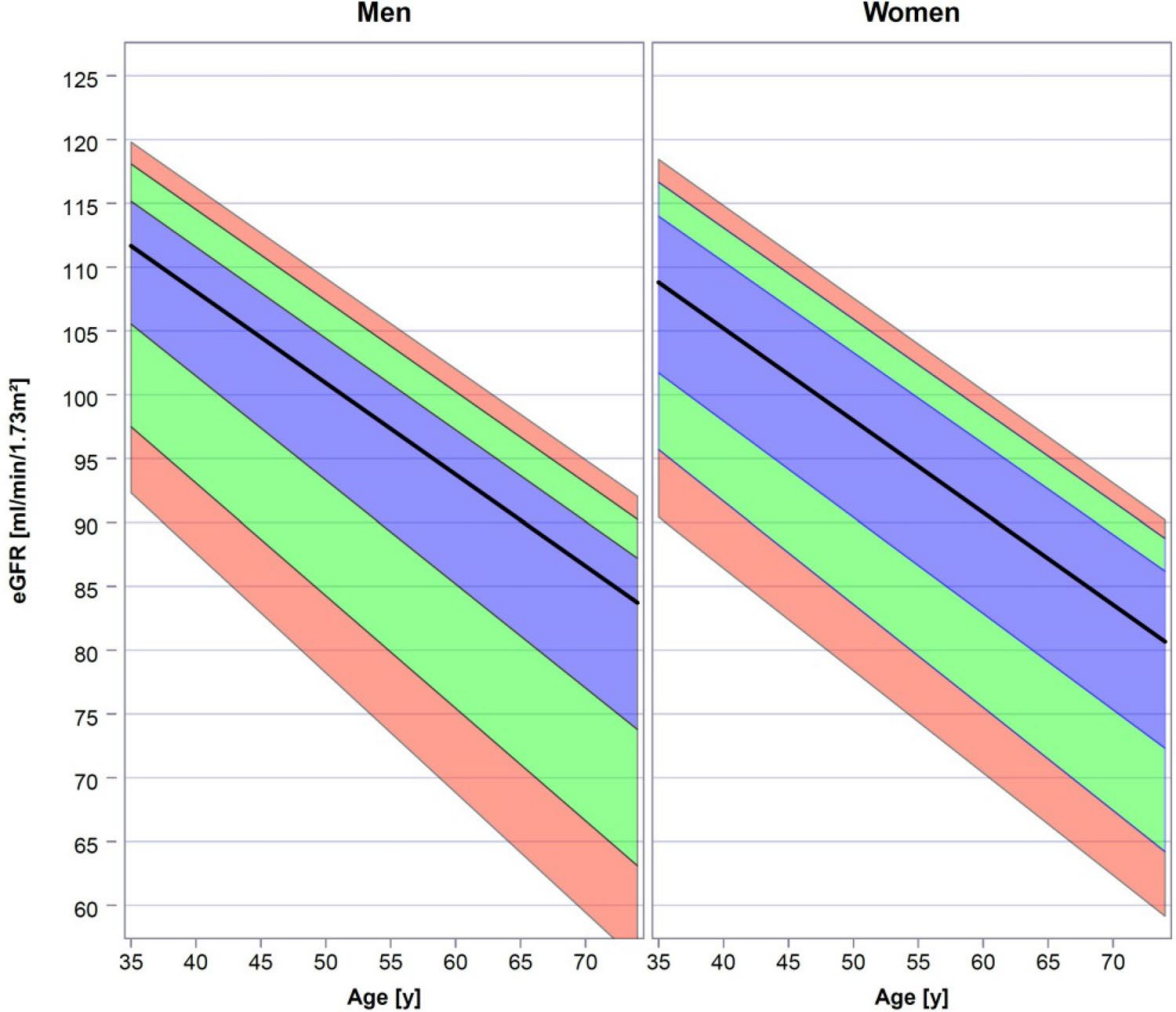
*Age dependent distribution of eGFR in men and women.* Median (black line) and interquartile range (blue area) are indicated. Green area indicates the ranges 10th–25th and 75th–90th percentiles, respectively. Red area indicates the ranges 5th–10th percentiles and 90th–95th percentiles. Data were obtained by quantile regression.

*eGFR and loss of eGFR in the population.* eGFR was calculated by the CKD-EPI equation from creatinine concentration and the demographic data at baseline and 5-year follow-up. The age dependent distribution of eGFR for both sexes obtained by quantile regression is shown in Fig. 1. As expected, in both sexes an age dependent decrease of eGFR is observed. In this cross-sectional dataset median eGFR decreases from approx. 110 ml/min/1.73 m^2^ at the age of 35 years to approx. 80–85 ml/min/1.73 m^2^ at 75 years in both men and women.

As a consequence, the proportion of participants with eGFR below the recommended threshold for chronic kidney disease of 60 ml/min/1.73 m^2^ increased with age. Table 1 summarizes the proportion of persons with eGFR below 75, 60, and 45 ml/min/1.73 m^2^ in the four age decades of our study population. Only individuals without albuminuria were included in this analysis.

**Table 1:** Age dependent proportion of persons with eGFR below 75, 60, and 45 ml/min/1.73 m^2^.

Sex	Age	eGFR < 75 ml/min/1.73 m^2^	eGFR < 60 ml/min/1.73 m^2^	eGFR < 45 ml/min/1.73 m^2^
Men	35–44	0.6%	0.0%	0.0%
	45–54	2.2%	0.1%	0.1%
	55–64	7.8%	0.7%	0.0%
	65–74	19.7%	4.5%	0.6%
Women	35–44	0.7%	0.0%	0.0%
	45–54	1.9%	0.1%	0.0%
	55–64	9.2%	1.1%	0.1%
	65–74	21.7%	3.4%	0.7%

**Figure 2:**
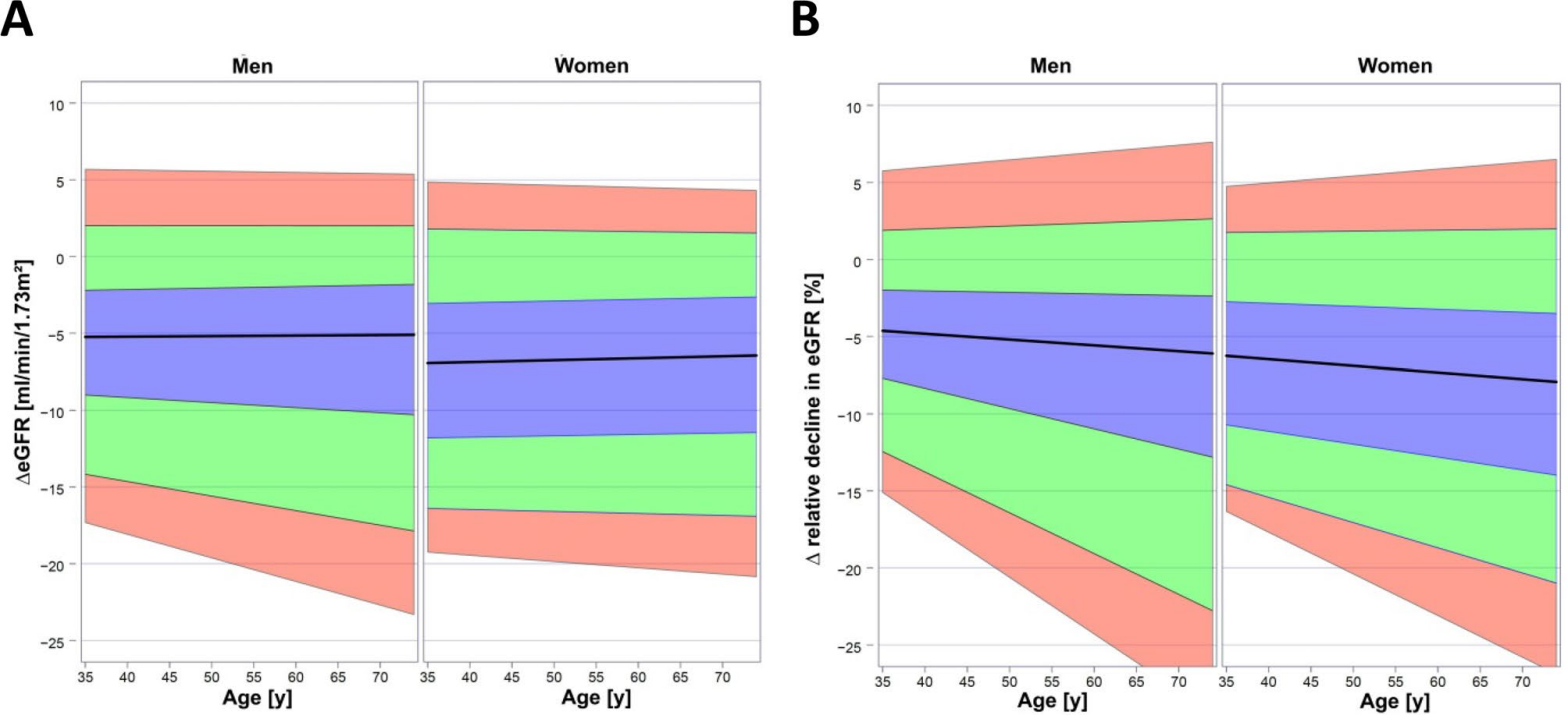
*Change in eGFR over the 5-year follow-up.* (**A**) Absolute change in eGFR. (**B**) Relative (%) change in eGFR. Median (black line) and interquartile range (blue area) are indicated. Green area indicates the ranges 10th–25th and 75th–90th percentiles, respectively. Red area indicates the ranges 5th–10th percentiles and 90th–95th percentiles. Data were obtained by quantile regression.

After 5 years of follow-up eGFR was calculated again and absolute and relative decline over the observation period were determined (Fig. 2A,B). In both sexes the dispersion of absolute and relative decline in eGFR increased with age. The increase of dispersion was predominantly related to a higher frequency of individuals with more rapid loss of eGFR in the older age groups. The median absolute loss of eGFR decreased slightly with age while the median relative loss of eGFR increased slightly with age. Since data from the 10-year follow-up were available for 3,282 participants, we also determined eGFR in this subgroup. Decline of eGFR continued over the second 5 year period (supplementary Fig. 1). However, the proportion of individuals with rapid decline, i.e. more than

3 ml/min/1.73 m^2^ per year or relevant increase, i.e. more than 1 ml/min/1.73 m^2^ per year was smaller than at the 5-year follow-up (supplementary table). This indicates that random intra-individual variations of creatinine had a greater effect after 5 years than after 10 years.

We have also calculated eGFR by a novel equation proposed by Pottel et al.^19^. While the results are very similar with a correlation coefficient of 0.77, median eGFR was approx. 5% lower than with CKD-EPI (90.1 vs. 94.9 ml/min/1.73 m^2^), and decline of eGFR per year was approx. 8% higher (median 6.38 vs. 5.91 ml/min/1.73 m^2^/5 years) (supplementary Fig. 2A–C).

**Figure 3:**
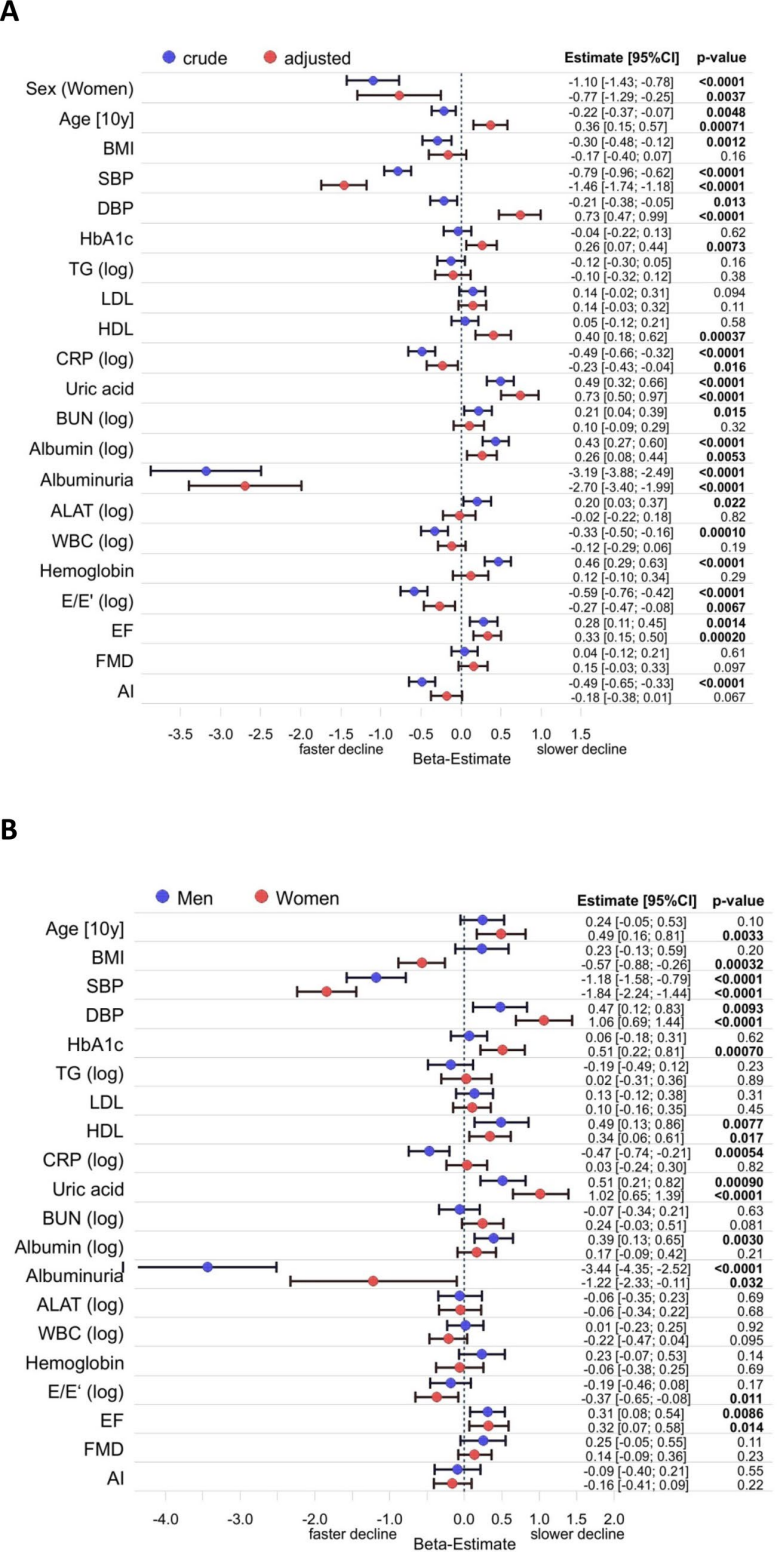
*Forest plot for variables affecting absolute change in eGFR over 5 years.* (**A**) Crude and fully adjusted data for the whole cohort. Linear regression was adjusted for age, sex and all variables listed. (**B**) Data for men and women adjusted for age and all variables listed. Regression coefficients (beta) for each variable are depicted for males and females with the corresponding p-value. They refer to a 1 SD change in the respective variable with the exception of age, sex and the presence of albuminuria. If data were log-transformed before analysis this is indicated by (log). AI–augmentation index; ALAT–alanine aminotransferase; BMI–body mass index, BUN–blood urea nitrogen; DBP–diastolic blood pressure; EF–ejection fraction; FMD–flow mediated dilatation; HDL–high density lipoprotein cholesterol; LDL–low density lipoprotein cholesterol; SBP–systolic blood pressure; TG–triglycerides; WBC–white blood cells.

*Factors associated to loss of eGFR.* We determined a broad spectrum of clinical and laboratory baseline characteristics of the study sample related to cardiovascular function, metabolism, renal function and others. These are summarized in Table 2. In a first step associations with absolute decline in eGFR were analyzed in the whole cohort (Fig. 3A,B). It should be noted here that in this exploratory analysis p-values indicated in the figure are considered as indicators of the potential relevance of these associations rather than as simple significances. When crude data were analyzed numerous factors were associated with the change in eGFR in men and women over the observation period of 5 years. After full adjustment several associations remained. Besides age and sex which are part of the CKD-EPI equation and may therefore be subject to confounding, several strong associations with substantial beta-estimates were found. Albuminuria was present in 6% of the cohort and was overall the factor with the strongest association with faster decline of eGFR in the fully adjusted model. Systolic blood pressure (SBP) had the next largest negative beta-estimate (i.e. greater decrease of eGFR per SD increase or presence of trait) of all factors analyzed. On the other hand an increase in diastolic blood pressure (DBP) was associated with a slower decline in eGFR. It should be noted though that this was only observed after adjustment, including adjustment for SBP. When the model did not include adjustment for SBP, DBP had a small negative beta-estimate of -0.23 (95% CI −0.42/−0.05; p = 0.012).

**Table 2:** *Baseline characteristics of the cohort.* Data are shown as median (interquartile range) for continuous variables and percentage (n) for discrete variables.

	All (12,381)	Men (6,349)	Women (6,032)
Sex (women)	48.7% (6032)	0%	100.0%
Age [y]	54.0 (45.0/64.0)	55.0 (46.0/64.0)	54.0 (45.0/63.0)
BMI [kg/ m^2^]	26.4 (23.8/29.7)	27.1 (24.8/30.0)	25.5 (22.8/29.3)
Obesity (n)	23.5% (2905)	24.8% (1573)	22.1% (1332)
Diabetes (n)	7.3% (900)	9.3% (589)	5.2% (311)
Hypertension (n)	49.3% (6104)	54.5% (3457)	43.9% (2647)
Dyslipidemia (n)	26.6% (3274)	36.4% (2285)	16.4% (989)
*Kidney function*			
Creatinine [mg/dl]	0.79 (0.71/0.89)	0.87 (0.80/0.96)	0.72 (0.67/0.78)
eGFR [ml/min/1.73 m^2^]	94.9 (86.5/102.9)	95.6 (87.6/103.7)	94.0 (85.5/102.0)
Albuminuria (n)	6.0% (732)	7.4% (461)	4.6% (271)
*Biomarkers*			
Cholesterol [mg/dl]	219 (193/246)	215 (189/240)	223 (197/251)
Triglycerides [mg/dl]	103 (77/145)	115 (85/161)	94 (71/126)
LDL-cholesterol [mg/dl]	137 (115/161)	137 (116/160)	137 (114/161)
HDL-cholesterol [mg/dl]	55.6 (46.0/67.0)	49.0 (42.0/57.0)	64.0 (54.0/74.6)
HbA1c [%]	5.5 (5.2/5.8)	5.5 (5.2/5.8)	5.5 (5.2/5.8)
CRP [mg/l]	1.5 (0.5/3.0)	1.4 (0.5/2.7)	1.6 (0.5/3.3)
Uric acid [mg/dl]	4.60 (3.70/5.70)	5.40 (4.70/6.30)	3.80 (3.14/4.60)
Urea nitrogen [mg/dl]	14.0 (11.0/16.0)	15.0 (12.0/17.0)	13.0 (11.0/15.0)
Albumin [g/l]	42.0 (40.0/44.0)	42.0 (40.0/44.0)	41.0 (39.0/44.0)
ALAT [U/l]	31 (25/41)	37 (29/47)	27 (22/33)
GGT [U/l]	24 (17/37)	30 (22/46)	19 (14/27)
Alkaline phosphatase [U/l]	64 (54/77)	64 (55/76)	64 (53/78)
Bilirubin [mg/dl]	0.64 (0.49/0.85)	0.71 (0.55/0.93)	0.57 (0.44/0.74)
White cell count [1/nl]	6.83 (5.79/8.15)	6.78 (5.70/8.07)	6.90 (5.87/8.20)
Red cell count [1/pl]	4.70 (4.44/4.98)	4.90 (4.66/5.14)	4.51 (4.30/4.73)
Haemoglobin [g/dl]	14.3 (13.5/15.2)	15.0 (14.4/15.7)	13.6 (13.0/14.2)
Homocystein [µmol/l]	11.0 (9.3/13.2)	12.0 (10.2/14.2)	10.1 (8.5/12.0)
*Cardiovascular function*			
Systolic blood pressure [mmHg]	129 (119/141)	132 (122/142)	126 (115/138)
Diastolic blood pressure [mmHg]	82.0 (76.0/88.5)	83.5 (78.0/89.5)	80.5 (74.5/87.0)
Ejection fraction [%]	63.5 (60.0/67.1)	63.0 (59.5/66.7)	64.2 (60.7/67.5)
E/E'-Ratio	7.08 (5.85/8.78)	6.89 (5.66/8.56)	7.29 (6.07/9.01)
Flow mediated dilatation [%]	7.34 (4.58/10.89)	6.11 (3.78/8.77)	9.20 (5.88/13.32)
Augmentation index [%]	13.90 (2.62/28.29)	8.18 (-1.74/20.26)	20.55 (9.16/35.64)
*Medications*			
Antidiabetics (n)	5.0% (618)	6.7% (420)	3.3% (198)
Diuretics (n)	4.1% (504)	4.0% (250)	4.2% (254)
Beta-Blockers (n)	15.7% (1919)	16.2% (1013)	15.1% (906)
Calcium antagonists (n)	6.4% (789)	7.5% (472)	5.3% (317)
Agents acting on RAS (n)	22.1% (2701)	25.7% (1610)	18.2% (1091)
Other antihypertensives (n)	0.8% (68)	0.8% (34)	0.8% (34)
Lipid modifying agents (n)	12.4% (1522)	15.0% (938)	9.8% (584)

Both impaired systolic (EF) and diastolic (E/E’) left ventricular function were associated with a greater decline in eGFR. Augmentation index (AI) as an indicator of arterial stiffness was associated with a greater decline in eGFR only in the crude model. After adjustment the p-value increased to 0.067. Endothelial function (FMD) did not show an association to eGFR change. Notably, BMI was only associated to the change in eGFR in the crude model but lost its association after adjustment. Another indicator of obesity, i.e. waist to height ratio was not associated neither in the crude nor the adjusted model (data not shown). Among biomarkers higher serum albumin and uric acid concentrations unexpectedly were strongly associated to a lesser decline in eGFR both in the crude and adjusted model. HDL-cholesterol and HbA1c were not associated with a lesser decline in the crude model but became significant after adjustment. Biomarkers associated with a greater decline of eGFR in the adjusted model were C-reactive protein (CRP) and white blood cell count (WBC). Total cholesterol, LDL-cholesterol, triglycerides, ALAT, urea nitrogen and hemoglobin were not associated to the change in eGFR after adjustment.

When the data were analyzed separately for both sexes, the majority of associations remained significant in both sexes. The association of albuminuria with decline of eGFR was stronger in men than in women. For diastolic dysfunction (E/E’) and albumin the association remained significant for only women or men, respectively, but the other sex showed the same trend. For HbA1c and CRP the associations remained only significant for women or men, respectively, while the other sex did not show any trend. It should be noted though, that all these factors had low to moderate β-estimates (< 0.5 ml/min per SD).

Since it has been reported that the effect of risk factors for loss of eGFR may be age associated^20^ we analyzed the interaction of the factors included in our study with age. There was only one significant interaction for HbA1c which changed the association of HbA1c with slower decline of eGFR in younger participants to faster decline in older participants (supplementary Fig. 3).

Recalculation of the data with the equation proposed by Pottel et al.^19^ did not result in relevant differences to these data with the only exception of age, which lost its association with decline of eGFR (supplementary Fig. 4A, B). Similarly, recalculation of the data without normalization to body surface area (BSA) did not change the association of risk factors with the rate of loss of eGFR (data not shown).

**Figure 4:**
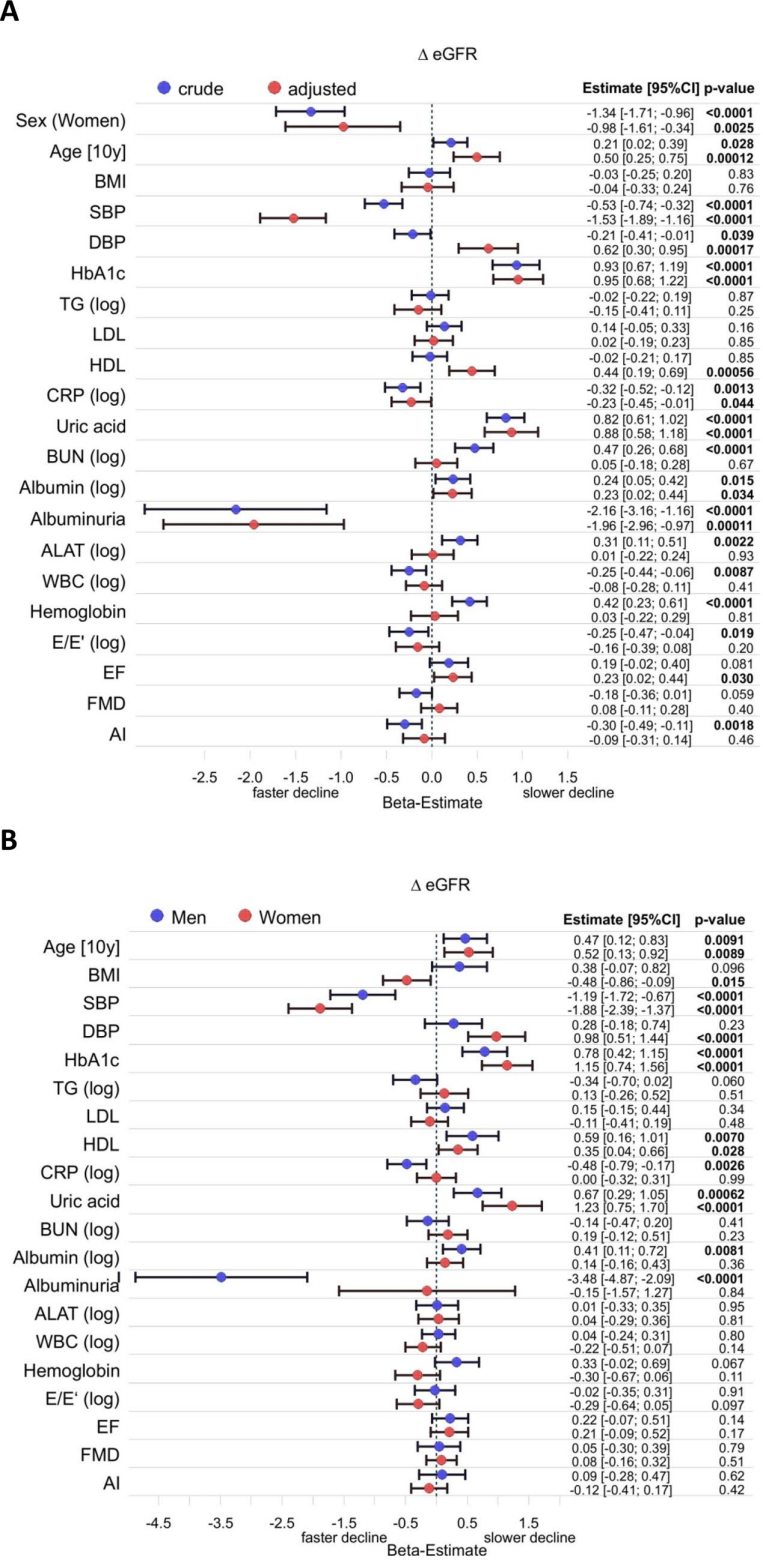
*Forest plot for variables affecting absolute change in eGFR in study participants who were not on antihypertensive, antidiabetic or lipid lowering medications.* (**A**) Crude and fully adjusted data for the whole cohort. Linear regression was adjusted for age, sex and all variables listed. (**B**) Data for men and women adjusted for age and all variables listed. Regression coefficients (beta) for each variable are depicted for males and females with the corresponding p-value. They refer to a 1 SD change in the respective variable with the exception of age, sex and the presence of albuminuria. If data were log-transformed before analysis this is indicated by (log). AI–augmentation index; ALAT–alanine aminotransferase; BMI–body mass index, BUN–blood urea nitrogen; DBP–diastolic blood pressure; EF–ejection fraction; FMD–flow mediated dilatation; HDL–high density lipoprotein cholesterol; LDL–low density lipoprotein cholesterol; SBP–systolic blood pressure; TG–triglycerides; WBC–white blood cells.

To analyze associations in more depth we excluded all patients who took diuretics, antihypertensives, antidiabetics, or lipid lowering medications. The remaining 3,307 study participants (Table 3) were analyzed in the same way as the whole cohort (Fig. 4). While most associations remained, HbA1c was now strongly associated with a lesser decline of eGFR in this selected subgroup. Due to the relatively small number of individuals with albuminuria (75 men and 38 women) confidence intervals for albuminuria were comparably large. In women there was no association of albuminuria with loss of eGFR. Otherwise, there were again no relevant sex differences. Again, recalculation with the alternative equation (supplementary figs. 5A, B) or without normalization to BSA (data not shown) did not result in relevant differences besides the loss of the association of eGFR loss with age.

**Table 3:** *Baseline characteristics of the participants without any regular medication.* Data are shown as median (interquartile range) for continuous variables and percentage (n) for discrete variables.

	All (3,307)	Men (2,122)	Women (1,185)
Sex (Women)	35.8% (1,185)	0% (0)	100.0% (1,185)
Age [y]	48.0 (42.0/56.0)	48.0 (42.0/56.0)	48.0 (42.0/56.0)
BMI [kg/ m^2^]	25.6 (23.3/28.4)	26.2 (24.1/28.9)	24.2 (22.0/27.1)
Obesity (yes)	16.0% (529)	18.2% (386)	12.1% (143)
Diabetes (yes)	1.2% (39)	1.2% (26)	1.1% (13)
Hypertension (yes)	26.9% (889)	31.5% (668)	18.6% (221)
Dyslipidemia (yes)	18.4% (605)	25.5% (536)	5.8% (69)
*Kidney function*			
Creatinine [mg/dl]	0.81 (0.73/0.90)	0.87 (0.80/0.94)	0.71 (0.66/0.76)
eGFR [ml/min/1.73 m^2^]	99.7 (91.5/106.7)	100.0 (92.3/107.2)	98.8 (90.3/106.0)
Albuminuria	3.5% (113)	3.6% (75)	3.3% (38)
*Biomarkers*			
Cholesterol [mg/dl]	217 (193/244)	218 (194/244)	216 (191/245)
Triglycerides [mg/dl]	96 (72/135)	107 (79/151)	81 (63/106)
LDL-cholesterol [mg/dl]	138 (118/162)	142 (122/164)	132 (111/157)
HDL-cholesterol [mg/dl]	54.0 (45.6/65.6)	50.0 (43.0/58.0)	65.0 (55.0/75.0)
HbA1c [%]	5.4 (5.1/5.6)	5.4 (5.1/5.6)	5.4 (5.1/5.6)
CRP [mg/l]	1.2 (0.5/2.2)	1.2 (0.5/2.2)	1.2 (0.5/2.2)
Uric acid [mg/dl]	4.7 (3.8/5.6)	5.3 (4.6/6.0)	3.5 (3.0/4.2)
Urea nitrogen [mg/dl]	13.0 (11.0/16.0)	14.0 (12.0/16.0)	12.0 (10.0/14.0)
Albumin [g/l]	42.0 (40.0/44.0)	42.0 (40.0/44.0)	42.0 (40.0/44.0)
ALAT [U/l]	32 (25/42)	36 (29/46)	26 (21/32)
GGT [U/l]	23 (16/35)	28 (20/41)	16 (13/22)
Alkaline phosphatase [U/l]	63 (53/76)	65 (55/76)	62 (52/75)
Bilirubin [mg/dl]	0.67 (0.52/0.89)	0.72 (0.56/0.95)	0.59 (0.45/0.78)
White cell count [1/nl]	6.65 (5.58/7.90)	6.54 (5.50/7.85)	6.83 (5.73/8.00)
Red cell count [1/pl]	4.79 (4.53/5.08)	4.96 (4.73/5.18)	4.51 (4.30/4.72)
Haemoglobin [g/dl]	14.6 (13.8/15.4)	15.1 (14.5/15.8)	13.6 (13.0/14.2)
Homocystein [µmol/l]	11.1 (9.4/13.3)	11.8 (10.2/14.0)	9.8 (8.3/11.6)
*Cardiovascular function*			
Systolic glood pressure [mmHg]	127 (117/137)	130 (121/139)	121 (111/132)
Diastolic blood pressure [mmHg]	82 (76/89)	84 (78/90)	79 (73/85)
Ejection fraction [%]	63.3 (60.0/66.7)	62.9 (59.6/66.3)	64.3 (60.8/67.2)
E/E'-Ratio	6.45 (5.45/7.77)	6.31 (5.32/7.62)	6.71 (5.70/8.03)
Flow mediated dilatation [%]	7.51 (4.66/11.09)	6.32 (4.02/9.03)	10.61 (6.78/14.49)
Augmentation index [%]	8.52 (-2.18/22.52)	3.91 (-5.44/15.78)	17.56 (5.91/33.32)

## Discussion

We present an analysis of determinants for the age dependent decline of GFR in a German cohort representative for the general population of Western Germany. The strength of our study is the large size of the population based study cohort and the broad spectrum of clinical and biochemical phenotypes available. Our study confirms and extends previous data. In some cases we observed differences to published data. Overall, eGFR declined by approx. 1 ml/min per year which is in line with previous data. This is associated with an increased percentage of persons over 65 years of age with eGFR < 60 ml/min/1.73 m^2^, i.e. with CKD stage G3a even when individuals with albuminuria are excluded (Table 1). This is in line with recent findings on kidney function in healthy elderly individuals^21^ and proposals for an age-adapted definition of CKD^6^.

Baseline factors associated most strongly with a more rapid decline of eGFR in our cohort, i.e. with the highest absolute beta-estimate, were albuminuria, SBP, reduced ejection fraction of the left ventricle (EF), and diastolic dysfunction as determined by E/E’. It should be noted that the beta-estimate refers to 1 SD change of the independent quantitative variables which may have different clinical implications depending on the variable. These associations were also observed in the subgroup of individuals without medications. WBC count showed a weak association with greater decline of GFR only in the whole group while in the medication free group this association was lost in the fully adjusted model. Factors associated with a lesser than average decline of eGFR were serum albumin, serum uric acid, and diastolic blood pressure. In the subgroup without medication also HbA1c was associated with lesser decline of eGFR.

SBP has been described in most previous studies as risk factor for an increased rate of decline of eGFR^22–26^. In our study SBP had consistently the highest absolute beta-estimate of all risk factors analyzed except albuminuria in the whole sample, the subsample without medication, and in both sexes. On the other hand, DBP was associated with lesser decline of eGFR after full adjustment. This was again the case for both sexes and also for the subsample without medication. This observation appeared surprising at first glance, but has been observed previously. Hirayama et al.^25^ described a lack of association of DBP with renal function decline. Also in another study the association of blood pressure with decline of GFR is mainly seen with SBP^26^. However, the association of DBP with faster decline of eGFR disappeared only after adjustment for SBP. This suggests that in persons with arterial hypertension a large pulse pressure, i.e. a relatively lower DBP is detrimental. This is also compatible with the previously reported association of pulse pressure with eGFR decline^27^. This might indicate that arterial stiffness with loss of elasticity and increased amplitude of blood pressure is a relevant factor which might explain the inverse relation of DBP and loss of eGFR. Indeed, in the whole cohort and the subgroup without medication an increased AI was associated with greater decline of eGFR in the crude data. In the fully adjusted model only a trend (p = 0.067) towards greater decline of eGFR was observed in the whole group. In the group without any medication AI had no association with change in eGFR (p = 0.40). These data show that while blood pressure is a key determinant for eGFR loss, its interaction with renal function is complex and may be modified by so far unidentified confounders.

A strong and consistent association of eGFR decline was observed with measures of left ventricular systolic (EF) and diastolic (E/E’) function in the whole cohort. Decreased left ventricular function was a strong predictor of faster decline in eGFR in both sexes. It has been previously shown in a cross-sectional community based study that eGFR is associated with several measures of left ventricular function^28^. We extend this finding now by showing that also the rate of eGFR decline is greater in persons with impaired systolic or diastolic left ventricular function at baseline. This underscores the relevance of the interaction between cardiac and renal function.

On the other hand endothelial function measured by FMD apparently had no effect on eGFR. After adjustment, endothelial function had neither in the whole group nor in the group without medication any association with the rate of decline of eGFR.

Another relevant finding of this study was the lack of association of eGFR decline with glycated hemoglobin in the whole cohort. Interestingly, in the crude model there was no association of HbA1c with eGFR decline. After full adjustment, HbA1c was associated with a slower decline of eGFR. Of all variables analyzed only HbA1c showed a strong interaction with age. While it was associated with slower loss of eGFR in participants younger than 55 years, it was associated with more rapid loss of eGFR in participants older than 55 years (supplementary Fig. 3). In the subgroup without medication, which was significantly younger than the whole cohort, HbA1c was associated in both models with a significantly slower decline of eGFR (β-estimate 0.93 and 0.94 respectively). Another major difference between the whole group and the medication-free group is the prevalence of diabetes (7.3% vs. 1.2%) which again is determined by age. This indicates that the inverse association of HbA1c with eGFR decline is limited to persons without diabetes. Our results are in contrast to some but not all other studies. As stated above the association of HbA1c and eGFR might be different in diabetics and non-diabetics. Furthermore, since we did not differentiate between participants with overt and incipient diabetes in this analysis, this observation might be related to the initial hyperfiltration observed in diabetic individuals. Also parameters of lipid metabolism, i.e. lipoproteins, did not show any significant association to eGFR decline with the exception of HDL-cholesterol which was associated with a slower decline of eGFR.

Limitations. GFR in our population was calculated rather than measured directly. This may introduce bias in particular with regard to age and sex which are part of the CKD-EPI formula. Data regarding systematic differences between eGFR and measured GFR in epidemiologic studies are scarce and controversial with regard to superiority of mGFR^29,30^. Another limitation is the availability of only two creatinine determinations at baseline and 5-year follow-up. Thus, random intra-individual variations of creatinine may have a significant effect on eGFR. Preliminary data from the 10-year follow-up support this assumption, but they also show that overall loss of eGFR continues at the same pace.

## Methods

### Study cohort

The Gutenberg Health Study (GHS) is a population-based, observational, prospective, single-center cohort study in the Rhein-Main region in Germany with 15,010 participants recruited between April 2007 and March 2012^31^. The sample was drawn randomly from the local registry offices in the City of Mainz and the adjacent District of Mainz-Bingen with a total population of approx. 400,000 people. Persons between 35 and 74 years of age were enrolled which represent about half of the total population. The sample was stratified 1:1 for sex and residence (urban and rural) and in equal strata for decades of age. Every participant underwent a comprehensive, standardized 5-h clinical investigation including among others anthropometric measurements, assessment of cardiovascular function, i.e. blood pressure, 12-lead electrocardiogram, transthoracic echocardiogram, analysis of vascular function, as well as computer-assisted interviews on cardiovascular risk factors, lifestyle, and socioeconomic status, and blood sampling. Participants were asked for their current medications which were classified according to ATC-codes. Details of the study protocol have been described previously^31,32^. All participants were invited for a follow-up visit after 5 years. The study was designed according to the tenets of the revised Helsinki declaration and the study protocol and sampling were approved by the Ethics Commission of the State Chamber of Physicians of Rhineland-Palatinate (reference no. 837.020.07, original vote: 22.3.2007, latest update: 20.10.2015). All participants gave informed written consent to laboratory analyses, clinical examinations, sampling of biomaterial and the use of data records for research purposes.

For the present study we analyzed data of 12,381 subjects who had completed the 5-year follow-up visit and validation of their study data. Of these 6,349 (51.3%) were male and 6,032 (48.7%) were female (Table 1).

### Definitions

Diabetes mellitus was defined as either a physician diagnosis of diabetes mellitus or a fasting blood glucose concentration of ≥ 126 mg/dL after at least an 8-h fasting period or a blood glucose level of ≥ 200 mg/dL. Dyslipidemia was assumed if there was a physician diagnosis of dyslipidemia or a low density lipoprotein/high-density lipoprotein ratio of ≥ 3.5. Arterial hypertension was defined as antihypertensive drug treatment in the previous two weeks, or a mean systolic blood pressure of ≥ 140 mm Hg, or a mean diastolic blood pressure of ≥ 90 mm Hg in the second and third measurement after 8 and 11 min at rest.

### Cardiovascular assessments

Cardiovascular status of the participants was assessed as published previously^32–36^. Briefly, echocardiography included standard two-dimensional measurements and continuous and pulse-wave Doppler measurements (iE33 echocardiography system, Philips Electronics, Amsterdam, The Netherlands). Measures for left ventricular systolic function (ejection fraction; LVEF) and diastolic function (E/E’) were determined as described^33,34^. Flow mediated dilatation (FMD) as a surrogate of endothelial function was measured in the brachial artery under resting conditions and after induction of reactive hyperemia by 5 min upper-arm occlusion as described in detail previously^35^. Arterial stiffness was assessed by digital volume plethysmography using an EndoPat 2000 device (Itamar Medical, Caesarea, Israel) as described previously^36^. The resultant augmentation index (AI) is automatically calculated.

### Laboratory analyses

Venous blood was collected in supine position while the subject was in fasting state (i.e. overnight fast, if subject was examined before 12 p.m. and > 5 h fast, if subject was examined after 12 p.m.). Serum creatinine was determined by a modified Jaffé method on Abbott Architect c8000 or c16000 systems (Abbott Diagnostics, Wiesbaden, Germany). The method is traceable to NIST SRM 967. Albuminuria was determined from a random urine specimen collected during the visit and was defined as > 30 µg albumin/mg creatinine. Urine albumin concentration was determined by nephelometry (Siemens, Eschborn, Germany). Urine creatinine was determined by a modified Jaffé method on Abbott Architect c8000 systems. From 238 participants no data on albuminuria were available. All other laboratory measurements were carried out in the central laboratory of the university medical center using reagents and analyzers from Abbott diagnostics. Glomerular filtration rate was estimated by the CKD-EPI equation (eGFR)^37^. For an additional sensitivity analysis eGFR was also calculated by a recently proposed novel equation^19^.

### Data management and statistical analyses

Descriptive statistics included medians with interquartile ranges (25th–75th percentile) for continuous variables. Discrete variables were presented as relative and absolute frequencies. The association of delta eGFR with determinants was analysed with multivariable linear regression model and presented as forest plots. The sex- and age-related quantiles were estimated using quantile regression, stratified by gender, with age as covariate. All statistical comparisons were two-tailed. Because of the explorative character of the analysis a significance threshold was not defined for p-values. Accordingly, the p-values should be interpreted as a continuous measure of statistical evidence. Statistical analysis was performed using R, version 3.6.0 (http://www.R-project.org).

## Supplementary Information

Supplementary Information.

## References

[1] Glassock RJ, Rule AD (2016). Aging and the kidneys: anatomy, physiology and consequences for defining chronic kidney disease. Nephron.

[2] Schmitt R, Melk A (2017). Molecular mechanisms of renal aging. Kidney Int..

[3] Stevens, P.E. & Levin. A. Kidney Disease: Improving Global Outcomes Chronic Kidney Disease Guideline Development Work Group Members. Evaluation and management of chronic kidney disease: synopsis of the kidney disease: improving global outcomes 2012 clinical practice guideline. *Ann. Intern. Med.* **158**, 825–830 (2013) 10.7326/0003-4819-158-11-201306040-00007 23732715

[4] Bash LD (2009). Defining incident chronic kidney disease in the research setting: The ARIC Study. Am. J. Epidemiol..

[5] Benghanem Gharbi, M. et al. Chronic kidney disease, hypertension, diabetes, and obesity in the adult population of Morocco: how to avoid "over"- and "under"-diagnosis of CKD. *Kidney Int*. **89**, 1363–1371 (2016) 10.1016/j.kint.2016.02.019 27165829

[6] Delanaye P (2019). CKD: A call for an age-adapted definition. J. Am. Soc. Nephrol..

[7] Bash LD, Erlinger TP, Coresh J, Marsh-Manzi J, Folsom AR, Astor BC (2009). Inflammation, hemostasis, and the risk of kidney function decline in the Atherosclerosis Risk in Communities (ARIC) Study. Am. J. Kidney Dis..

[8] Shankar A (2011). Markers of inflammation predict the long-term risk of developing chronic kidney disease: a population-based cohort study. Kidney Int..

[9] Halbesma N (2011). Development and validation of a general population renal risk score. Clin. J. Am. Soc. Nephrol..

[10] Tin A (2015). Results from the Atherosclerosis Risk in Communities study suggest that low serum magnesium is associated with incident kidney disease. Kidney Int..

[11] Liu G (2016). Elevated plasma tumor necrosis factor-α receptor 2 and resistin are associated with increased incidence of kidney function decline in Chinese adults. Endocrine.

[12] Rebholz CM (2016). Relationship of the American Heart Association's Impact Goals (Life's Simple 7) with risk of chronic kidney disease: Results from the Atherosclerosis Risk in Communities (ARIC) Cohort Study. J. Am. Heart Assoc..

[13] Halbesma N (2008). Gender differences in predictors of the decline of renal function in the general population. Kidney Int..

[14] Hiramoto JS (2012). Inflammation and coagulation markers and kidney function decline: the Multi-Ethnic Study of Atherosclerosis (MESA). Am. J. Kidney Dis..

[15] Grubbs V (2014). Body mass index and early kidney function decline in young adults: a longitudinal analysis of the CARDIA (Coronary Artery Risk Development in Young Adults) study. Am. J. Kidney Dis..

[16] Sedaghat S (2016). von Willebrand factor, ADAMTS13 Activity, and decline in kidney function: A population-based cohort study. Am. J. Kidney Dis..

[17] Tin A (2015). Hemostatic factors, APOL1 risk variants, and the risk of ESRD in the atherosclerosis risk in communities study. Clin. J. Am. Soc. Nephrol..

[18] Grams ME (2016). Race, APOL1 Risk, and eGFR Decline in the General Population. J. Am. Soc. Nephrol..

[19] Pottel H (2021). Development and validation of a modified full age spectrum creatinine-based equation to estimate glomerular filtration rate. Ann. Intern. Med..

[20] Toyama T (2020). Age differences in the relationsships between risk factors and loss of kidney function: a general population cohort study. BMC Nephrol..

[21] Eriksen BO (2020). GFR in healthy aging: an individual participant data meta-analysis of iohexol clearance in European population-based cohorts. J. Am. Soc. Nephrol..

[22] Vupputuri S (2003). Effect of blood pressure on early decline in kidney function among hypertensive men. Hypertension.

[23] Kronborg J, Solbu M, Njolstad I, Toft I, Eriksen BO, Jenssen T (2008). Predictors of change in estimated GFR: a population based 7-year follow-up from the Tromso study. Nephrol. Dial. Transplant..

[24] Rifkin DE (2013). Blood pressure components and decline in kidney function in community-living older adults: the cardiovascular health study. Am. J. Hypertens..

[25] Hirayama A (2015). Blood pressure, proteinuria, and renal function decline: associations in a large community-based population. Am. J. Hypertens..

[26] Eriksen BO (2017). Blood pressure and age-related GFR decline in the general population. BMC Nephrol..

[27] Peralta, C.A. et al. Association of pulse pressure, arterial elasticity, and endothelial function with kidney function decline among adults with estimated GFR >60 mL/min/1.73 m^2^: the Multi-Ethnic Study of Atherosclerosis (MESA). *Am. J. Kidney Dis.* **59**, 41–49 (2012) 10.1053/j.ajkd.2011.08.015 PMC3242889 22000727

[28] Nerpin E (2014). The association between glomerular filtration rate and left ventricular function in two independent community-based cohorts of elderly. Nephrol. Dial. Transpl..

[29] Mathisen UD (2011). Estimated GFR associates with cardiovascular risk factors independently of measured GFR. J. Am. Soc. Nephrol..

[30] Ku E (2016). Change in measured GFR versus eGFR and CKD outcomes. J. Am. Soc. Nephrol..

[31] Wild PS (2012). The Gutenberg Health Study. Bundesgesundheitsblatt Gesundheitsforschung Gesundheitsschutz.

[32] Schnabel RB (2012). Multiple endothelial biomarkers and noninvasive vascular function in the general population: The Gutenberg Health Study. Hypertension.

[33] Wild PS (2010). Distribution and categorization of left ventricular measurements in the general population: Results from the population-based Gutenberg-Heart Study. Circ. Cardiovasc. Imaging.

[34] Schwarzl M (2016). Risk factors for heart failure are associated with alterations of the LV end-diastolic pressure-volume relationship in non-heart failure individuals: data from a large-scale, population-based cohort. Eur. Heart J..

[35] Schnabel RB (2011). Non-invasive vascular function measurement in the community: Cross-sectional relations and comparison of methods. Circ. Cardiovasc. Imaging.

[36] Panova-Noeva M (2017). Mean platelet volume and arterial stiffness - clinical relationship and common genetic variability. Sci. Rep..

[37] Levey, A.S et al. A new equation to estimate glomerular filtration rate. *Ann. Intern. Med.* **150**, 604–612 (2009) 10.7326/0003-4819-150-9-200905050-00006 PMC2763564 19414839

