# Supplementary material for: Distribution of estimated glomerular filtration rate and determinants of its age dependent loss in a German population-based study
Source: Sci Rep. 2021 May 13;11:10165. doi: 10.1038/s41598-021-89442-7 (PMC8119940; doi:10.1038/s41598-021-89442-7)

Supplementary data

Distribution of Estimated Glomerular Filtration Rate and Determinants of its Age Dependent Loss in a German Population-Based Study.

Thomas Waas, MD, Andreas Schulz, PhD Johannes Lotz, MD, Heidi Rossmann, MD, Norbert Pfeiffer, MD, Manfred E. Beutel, MD, Irene Schmidtmann, PhD, Thomas Münzel, MD, Philipp S. Wild, MD, Karl J. Lackner, MD

Supplementary table: Rate of annual change of eGFR at 5-year and 10-year follow-up in a subgroup of 3,282 participants who already completed 10-year follow-up.

	**5-year follow-up**		**10-year follow-up**	
**Δ eGFR [ml/min{1.73m^2/year]**	**Men [%] n = 1,682**	**Women [%] n = 1,600**	**Men [%] n = 1,682**	**Women [%] n = 1,600**
< -3	11.2	16.6	5.8	4.1
-1 to -3	42.4	48.9	52.6	66.6
-1 to +1	41.4	28.2	41.2	28.7
> +1	4.9	6.2	0.4	0.5

Suppl. fig. 1: Distribution of eGFR in the subgroup of participants who already completed the 10-year follow-up.

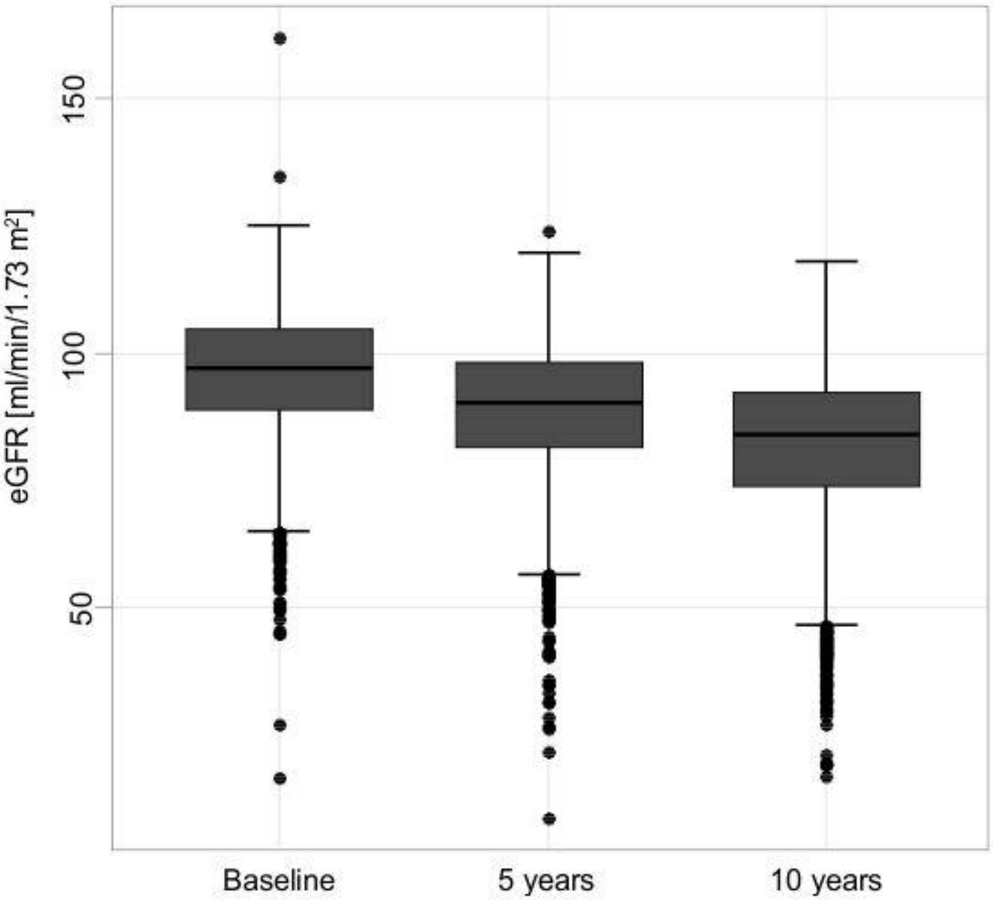

Suppl. fig. 2: eGFR and change of eGFR were recalculated with the equation proposed by Pottel et al. [19]. (A) Age dependent distribution of eGFR in men (left) and women (right). (B) Absolute change in eGFR over the 5-year follow-up. (C) Relative (%) change in eGFR. Median (black line) and interquartile range (blue area) are indicated. Green area indicates the ranges 10th-25th and 75th-90th percentiles, respectively. Red area indicates the ranges 5th-10th percentiles and 90th-95th percentiles. Data were obtained by quantile regression.

A

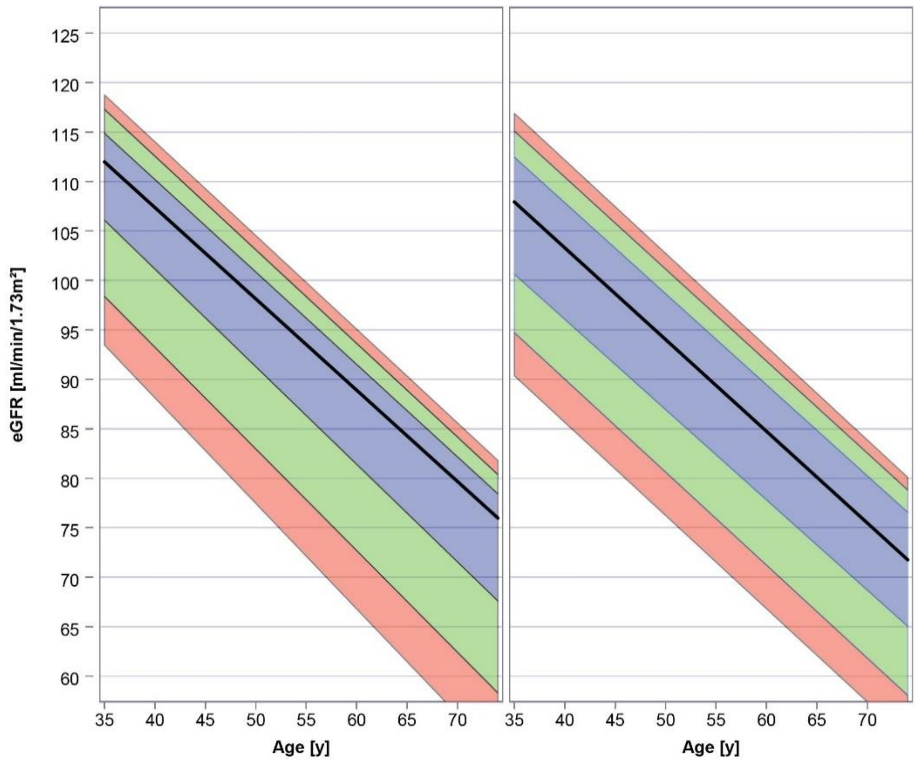

B

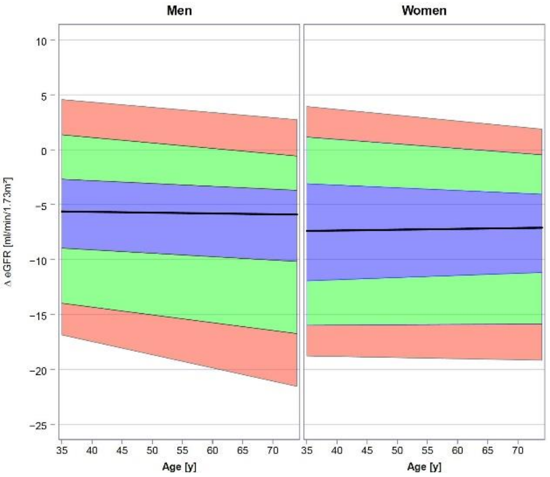

C

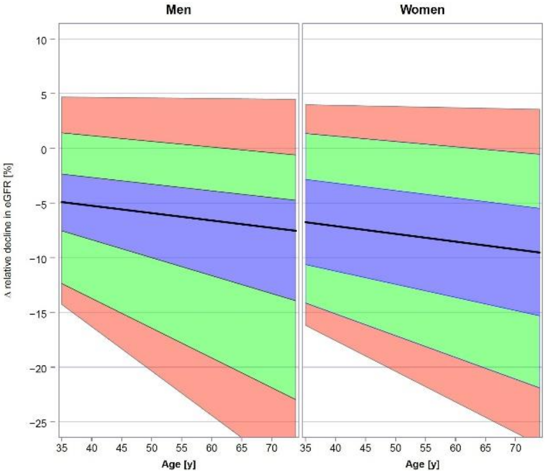

Suppl. fig. 3: Age dependency of the association of HbA1c with change in eGFR over 5 years. Black line represents the beta-estimate. Shaded area is the 95% confidence interval. Negative value of beta indicates association with more rapid loss of eGFR.

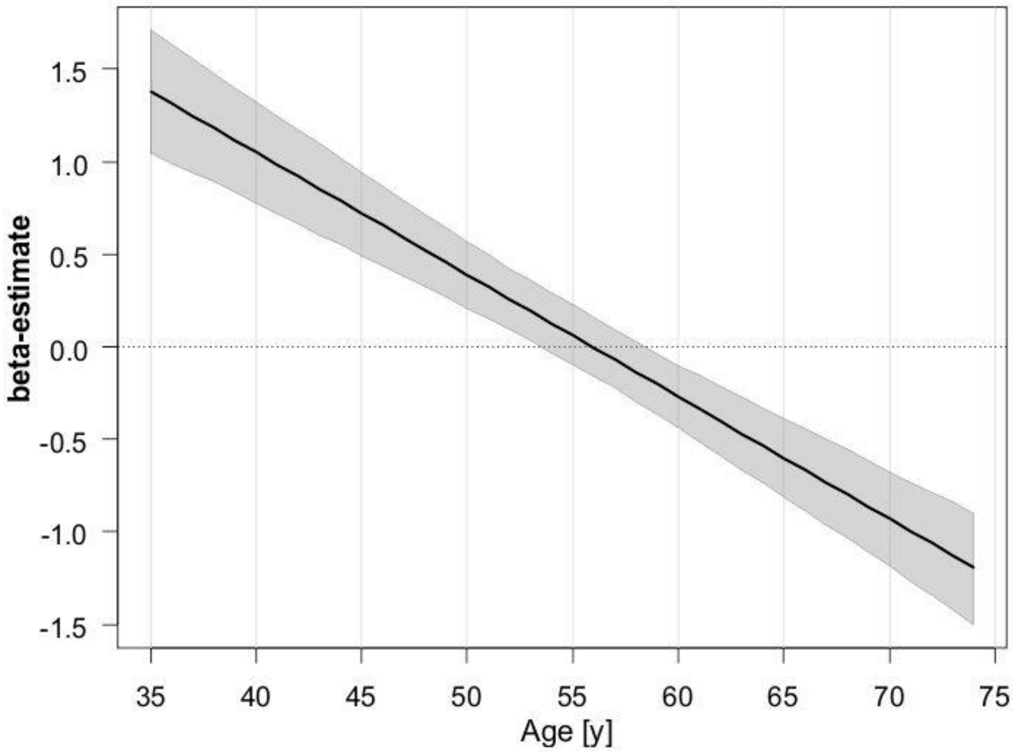

Suppl. fig. 4: *Forest plot for variables affecting absolute change in eGFR over 5 years.* (A) Crude and fully adjusted data for the whole cohort. Linear regression was adjusted for age, sex and all factors listed. (B) Data for men and women adjusted for age and all variables listed. Regression coefficients (beta) for each factor are depicted for males and females with the corresponding p-value.

A

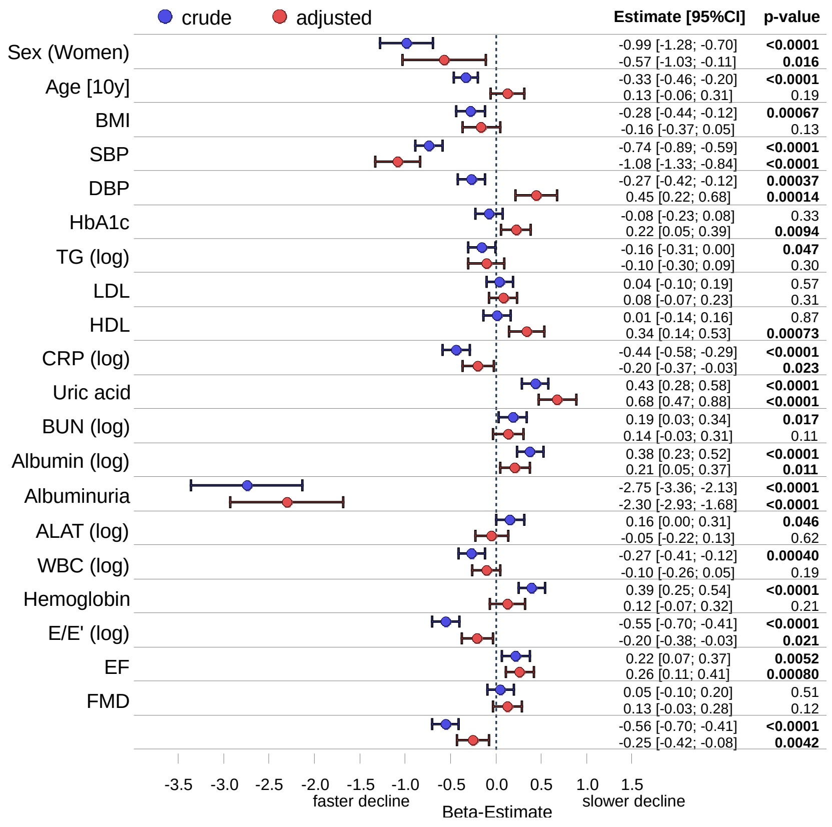

B

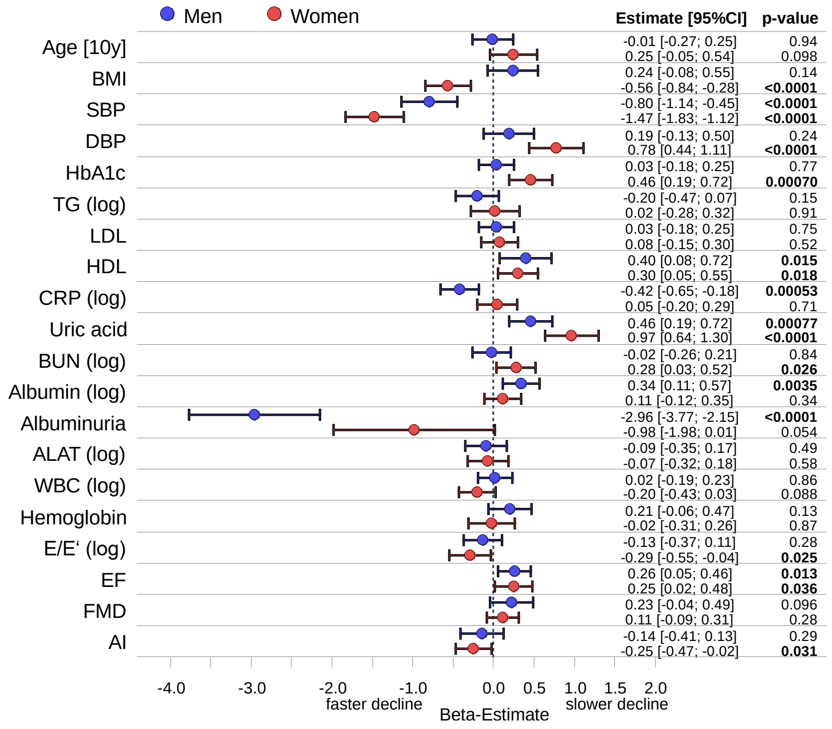

Suppl. fig. 5: *Forest plot for variables affecting absolute change in eGFR in study participants who were not on antihypertensive, antidiabetic or lipid lowering medications..* (A) Crude and fully adjusted data for the whole cohort. Linear regression was adjusted for age, sex and all factors listed. (B) Data for men and women adjusted for age and all variables listed. Regression coefficients (beta) for each factor are depicted for males and females with the corresponding p-value.

A

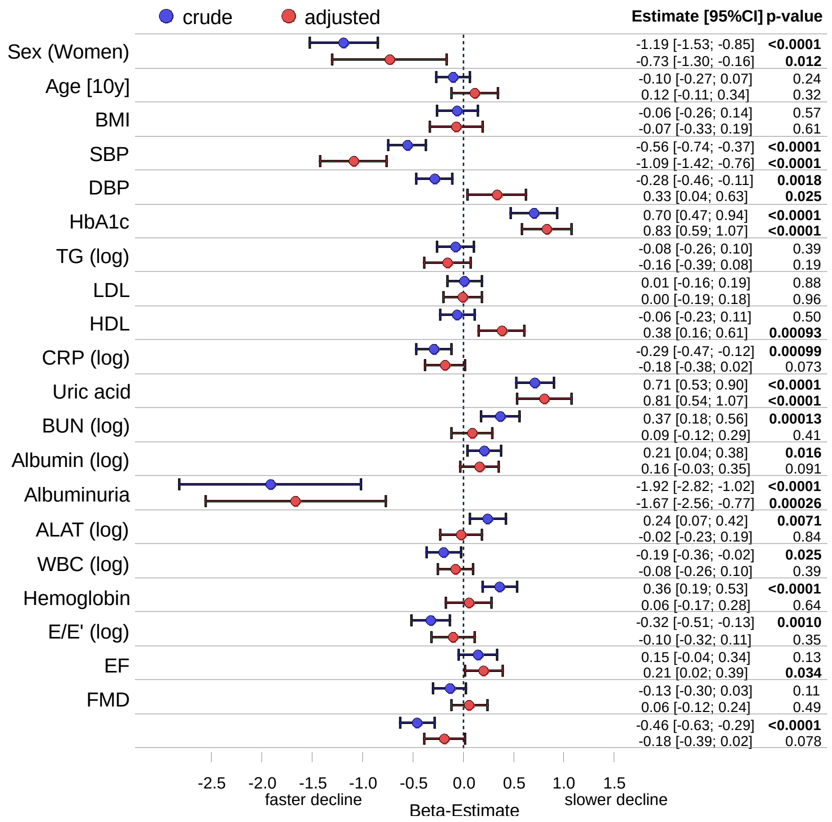

B

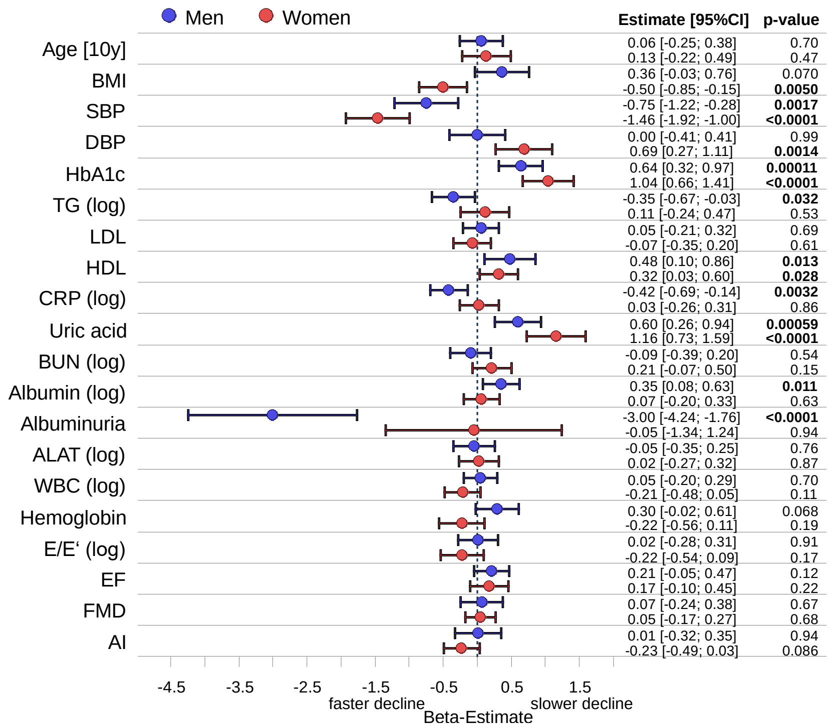

Supplement: Supplementary file 1 — Supplementary Information. [file 41598_2021_89442_MOESM1_ESM.pdf]
